# Chemical profiles of birch and alder bark by ambient mass spectrometry

**DOI:** 10.1007/s00216-019-02171-9

**Published:** 2019-10-23

**Authors:** Riikka-Marjaana Räsänen, Juha-Pekka Hieta, Juha Immanen, Kaisa Nieminen, Raisa Haavikko, Jari Yli-Kauhaluoma, Tiina J. Kauppila

**Affiliations:** 1 Drug Research Program, Division of Pharmaceutical Chemistry and Technology, Faculty of Pharmacy, P.O. Box 56, FI-00014 University of Helsinki, Helsinki, Finland; 2 Natural Resources Institute Finland (Luke), Production Systems, Plant Genetics, Viikinkaari 1, FI-00790 Helsinki, Finland; 3 Finnish Institute for Verification of the Chemical Weapons Convention (VERIFIN), Department of Chemistry, P.O. Box 55, FI-00014 University of Helsinki, Helsinki, Finland

**Keywords:** Triterpenoid, Bark, Desorption atmospheric pressure photoionization, Laser ablation atmospheric pressure photoionization, Ambient mass spectrometry, Mass spectrometry imaging

## Abstract

Desorption atmospheric pressure photoionization (DAPPI) is an ambient mass spectrometry (MS) technique that allows the analysis of both polar and nonpolar compounds directly from the surfaces of various sample types. Here, DAPPI was used to study the chemical profiles in different parts of birch and alder tree barks. Four distinct fractions of *Betula pendula* (silver birch) bark were collected from three different developmental stages of the stem, after which the chemical profiles of the different tissue types were measured. Of special interest were triterpenoids, a class of important defensive substances, which are found in the bark of the silver birch. Additionally, the chemical profiles of lenticels and the surrounding surfaces in the phellem of *B*. *pendula* (silver birch), *Alnus glutinosa* (black alder), and *Alnus incana* (gray alder) were screened with DAPPI. Another ambient MS technique, laser ablation atmospheric pressure photoionization (LAAPPI), was further used for the mass spectrometry imaging of lenticels on the *B*. *pendula* phellem. All the studied birch bark fractions showed individual chemical profiles in DAPPI. The mass spectra from the young apical stem and the transition zone resembled each other more than the mature stem. Instead, the phellem was found to contain a high amount of triterpenoids in all the developmental stages of the stem. The most intense peaks in the DAPPI mass spectra of the birch bark fractions were those of betulin and lupeol. Betulinic and betulonic acid peaks were intense as well, and these compounds were detected especially in the lenticels of the tree samples.

**Electronic supplementary material:**

The online version of this article (10.1007/s00216-019-02171-9) contains supplementary material, which is available to authorized users.

**Figure:**
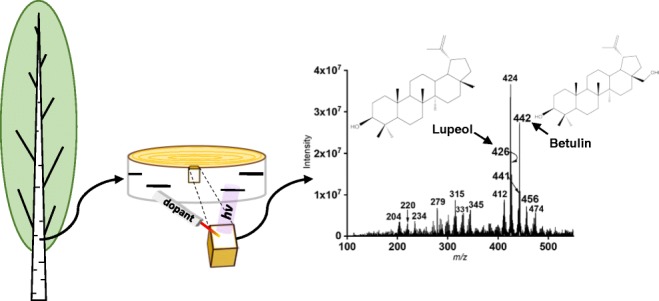
Graphical abstract

## Introduction

**Fig. 1:**
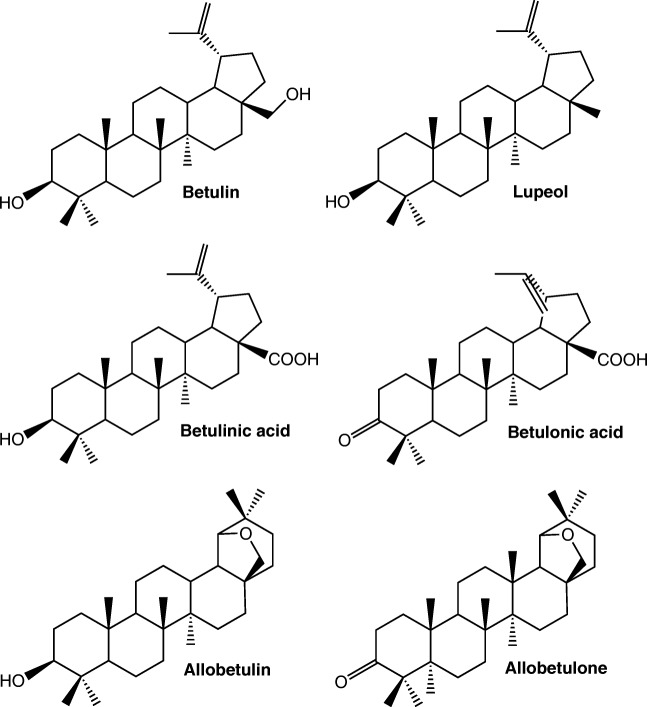
Structures of triterpenoids used in this study

**Fig. 2:**
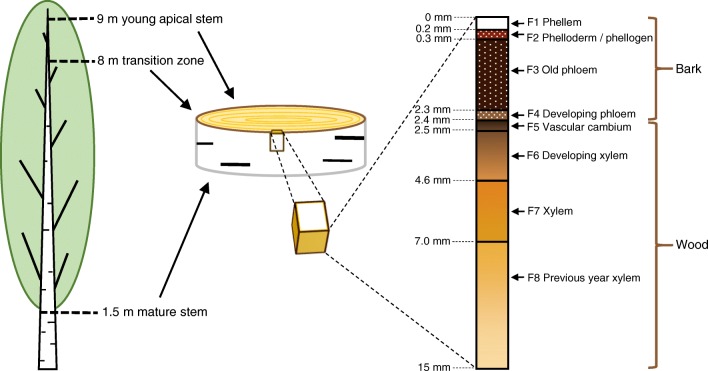
A schematic picture of the sampled fractions across vascular cambium of the *B*. *pendula* stem, tree samples, and the different fractions in the stem

It has been reported that over 50% of currently sold pharmaceuticals originate from natural products or naturally occurring compounds or their derivatives synthesized in the laboratory [1]. One natural compound that has proved to have interesting bioactivities is the triterpenoid betulin, and especially its natural derivatives like betulinic acid [2–4]. These compounds have been used as scaffolds in various drug development projects that aim at treatments of, e.g., bacterial inflammations [5] and cancer [6]. The structures of betulin and some other triterpenoids are presented in Fig. 1. In nature, betulin and its derivatives exist abundantly in the bark of *Betula* spp., especially in *Betula pendula* (silver birch) [4], but they are also found in the bark of many *Alnus* spp. [7, 8] which belong to the same family of Betulaceae. Bark is the outermost layer of the tree, and it consists of fractions with distinct tissue types. A schematic picture of the *B*. *pendula* bark structure containing four fractions (phellem, phelloderm/phellogen, old phloem, and developing phloem) is presented in Fig. 2. The color and the roughness of the bark surface are diverse for different tree species, and the appearance of the bark is also affected by lenticels, channels that enable the gas exchange from the surrounding atmosphere to the inner structures of the tree. Betulin and its derivatives are located mainly in the phellem, which is the outermost fraction of the bark [9].

Plants produce a vast and diverse array of metabolites and recent years have seen a surge of interest in their biotechnological potential. Complex plant samples containing plant metabolites are traditionally analyzed by multistep analytical techniques, such as gas chromatography (GC) or liquid chromatography (LC) separation coupled with mass spectrometry (MS) detection [10]. These methods require careful sample pretreatment with often several time-consuming steps, which increase the total analysis time remarkably. Additionally, large sample pieces are required for the pretreatment, and therefore small changes in the spatial distribution of the metabolites in the sample will be lost. For solid samples like plant leafs [11], fast surface sampling and analysis by ambient MS is also feasible. In ambient MS, the compounds are sampled directly from the surface in atmospheric pressure conditions and detected by MS [12]. Ambient MS techniques can be used for the rapid screening of compounds from the surfaces of, e.g., plants, tablets, or dried blood spots without prior sample preparation [13]. In addition, ambient MS makes possible the determination of the spatial distribution of different analytes in the sample, as in, e.g., mass spectrometry imaging of animal tissues [14], or plant metabolites [15].

**Fig. 3:**
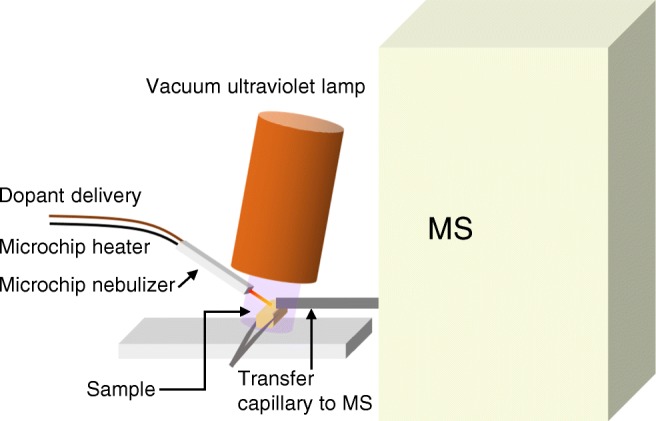
A schematic picture of the DAPPI technique and the sampling procedure

Desorption atmospheric pressure photoionization-mass spectrometry (DAPPI-MS) is an ambient MS technique, which can be used for the efficient detection of both polar and nonpolar compounds [16]. In DAPPI, the sampling surface is exposed to a hot solvent spray, which causes thermal desorption of the compounds from the surface (Fig. 3). The vaporized compounds are ionized through a series of reactions initiated by photons emitted from a vacuum ultraviolet (VUV) lamp, and the formed ions are directed to the MS for analysis. The ionization process in DAPPI has been reported to be similar to that in atmospheric pressure photoionization (APPI) [17]. The hot solvent spray acts as a dopant, which participates in the ionization process and enhances the ionization efficiency. The selection of the right type of dopant for the experiment is important. Earlier DAPPI studies have shown that acetone and toluene are efficient DAPPI dopants [13, 16, 17]. When toluene is used as a dopant, the formed analyte ions will be mainly molecular ions or protonated molecules, and when acetone is used, protonated molecules are formed. In plant analysis, DAPPI has been used to analyze cannabinoids from *Cannabis sativa* blooms [18], cathine from *Catha edulis* leaves [19], neonicotinoids from rose leafs [20], metabolites from *Peucedanum palustre* leafs [11], and α-tocopherol (vitamin E) from almond tree seeds [13]. Like APPI, also DAPPI is a very sensitive ionization technique for low polarity compounds [13, 16, 17, 21], which is beneficial in the plant analysis, since many plant metabolites have low polarities.

Another ambient MS technique based on photoionization is laser ablation atmospheric pressure photoionization (LAAPPI) [22]. In LAAPPI, the analytes are ablated from the sample surface with infrared (IR) laser and ionized through photoionization reactions. Thanks to the laser ablation, LAAPPI achieves an excellent spatial resolution of less than 50 μm [23], and therefore it is feasible for mass spectrometry imaging (MSI). Similarly to DAPPI, LAAPPI is suitable for both polar and nonpolar compounds, and thus far it has been used for the analysis of *Salvia officinalis* (sage) leafs [24, 25], *Citrus aurantium* (sour orange) leafs [22], microbial biofilms [25], and mouse brain tissue [23].

In this study, ambient MS techniques DAPPI and LAAPPI were applied to the direct analysis of compounds from bark samples. DAPPI-MS was used to distinguish the chemical profiles of four distinct bark fractions of *B*. *pendula*, with focus in triterpenoids of low polarity. In addition, DAPPI-MS was applied to a comparative study of bark surfaces and lenticels of *B*. *pendula* (silver birch), *Alnus glutinosa* (black alder), and *Alnus incana* (gray alder). Finally, LAAPPI-MSI was used to study the lenticels of *B*. *pendula*.

## Materials and methods

### Chemicals and samples preparation

HPLC grade acetone, methanol, and toluene were purchased from Sigma-Aldrich (St. Louis, MO, USA). Chloroform (99.2%) AnalaR NORMAPUR was from VWR (Briare, France). Triterpene standards betulin, betulinic acid, betulonic acid, allobetulin, and allobetulone were synthesized at the Division of Pharmaceutical Chemistry, Faculty of Pharmacy, University of Helsinki, and the synthesis procedure has been described elsewhere [26–28]. The standards were used as such without further purification. Lupeol (≥ 94%) was purchased from Sigma-Aldrich. For optimization of DAPPI-MS parameters, stock solutions of allobetulin and allobetulone were prepared in methanol with 9% of chloroform, and the other triterpenoid stock solutions in methanol. Structures of the triterpenoid standards are presented in Fig. 1.

Solid pieces of *B*. *pendula* bark from an adult tree were collected from three different developmental stages of the stem (Fig. 2) with characteristic bark surface colors: mature stem (white), transition zone (gray/light-brown), and young apical stem (brown). The samples were tangentially cryosectioned, as mentioned in [9], from each developmental stage. The four distinct fractions of birch bark; phellem, phelloderm/phellogen, old phloem, and developing phloem (Fig. 2), which are named F1, F2, F3, and F4, respectively, were analyzed with DAPPI-MS. Before the analysis, the outer-most layer was peeled off from fractions F2–F4. F1 fractions from the mature *B*. *pendula* stem were also studied with LAAPPI-MSI. For *A*. *glutinosa* and *A*. *incana*, the bark samples were cut from the mature stem (height 1.5 m) of the trees, and only fraction F1 was studied.

### DAPPI-MS

In DAPPI-MS (Fig. 3), hot solvent spray was produced by a microchip heated nebulizer [29]. The heating was controlled by ISO-TECH programmable power supply, 603 (Thurlby-Thanders Instruments Ltd., Huntington, England). The nebulizer gas (N_2_) flow rate, 180 mL min^−1^, was controlled with a mass flow controller (Aalborg, Orangeburg, NY, USA). The mass spectrometer (Agilent 6330 ion trap mass spectrometer, Agilent Technologies, Waldbronn, Germany) inlet was equipped with a capillary extension. The microchip heated nebulizer was aligned directly parallel to the MS inlet. The dopant jet impact angle was approximately 45°. The distances of the microchip nozzle from the sample surface, and the sample spot from the capillary extension inlet, were 3–4 and 1 mm, respectively. The vertical distance of the sample plate from the inlet was 1 mm. The VUV PKR 100 lamp (Heraeus Noblelight, Cambridge, UK) with 10.0 and 10.6 eV (minor portion) photon energy was placed directly above the microchip nozzle, the sample spot, and the capillary extension inlet. The spray solvent was pumped with a syringe pump model Harvard apparatus Pump 11 Elite (Harvard, Holliston, MA, USA) at a flow rate of 10 μL min^−1^. The tree samples were held in front of the MS inlet with tweezers, and they were exposed to the hot solvent jet for eight seconds. The MS data acquisition range was *m*/*z* 100–600, and the data were collected in positive ion mode.

The MS parameters for the DAPPI measurements were optimized for the triterpene compounds using direct infusion μAPPI [30]. For optimization10 μM solutions of the triterpenes in acetone and toluene were prepared, and the ionization efficiency of the compounds was studied in positive and negative ion modes. The sample solution flow rate was 10 μL min^−1^, and the nebulizer gas flow rate was 180 mL min^−1^.

### LAAPPI-MSI

F1 fractions of *B*. *pendula* bark from mature stem were imaged with LAAPPI-MSI [22]. In LAAPPI, a microscopic sample volume is ablated using a focused IR laser beam. As a result, tissue particles and biomolecules are transported to the gas-phase where they encounter a hot solvent jet sprayed by a microchip heated nebulizer [29]. The hot solvent jet dissolves tissue particles and biomolecules after which they can be ionized by a VUV lamp through photoionization reactions. Since the ionization principal in LAAPPI is similar to APPI and DAPPI reactions, LAAPPI is a suitable ionization method for both polar and nonpolar compounds. Moreover, LAAPPI is also applicable for MSI as the acquired mass spectra can be combined to the predetermined sample coordinates. In MSI measurement, the selected sample area is analyzed spot-by-spot after which heat map images are created to show distributions of compounds in the imaged sample [31].

A schematic picture of the LAAPPI-MSI setup is presented in the Electronic Supplementary Material (ESM) in Fig. S1. The setup consisted of (1) an optical setup based on a mid-IR laser (IR Opolette HE 2940 nm, OPOTEK, Carlsbad, CA, USA) for ablating the sample with a 400 μm sampling spot diameter; (2) a movable sample holder for rastering the sample surface spot-by-spot; (3) a microchip heated nebulizer [29] for producing the hot solvent jet for vaporization of the ablated projectiles, and to supply dopant for more efficient ionization; and (4) a VUV lamp, similar to that in the DAPPI-MS experiments to initiate the ionization. The mass spectra were collected by using an Agilent 6410 Triple Quadrupole mass spectrometer (Agilent Technologies, Santa Clara, CA, USA) with settings applied for focused measurements of betulin and its derivatives. Thus, the MS data acquisition range was set to *m*/*z* 400–500, and the data were collected in positive ion mode.

## Results and discussion

### Ionization of the triterpenoids in μAPPI

Ionization of the triterpenoid standards in APPI was studied with direct infusion μAPPI. In addition, the MS parameters were optimized for the DAPPI-MS measurements. Toluene and acetone were tested as dopants in both positive and negative ion modes. Table 1 summarizes the main ions observed for the triterpenoids. Fragments with intensity over 20% of the main peak are also presented.

**Table 1:** The main ions of triterpenoid standards in direct infusion μAPPI-MS with toluene and acetone dopants. The most abundant ions are presented for each compound (intensity over 20% of the main peak). The sample concentrations were 10 μM, and sample solution flow rate was 10 μL min^−1^. The data was collected in both positive and negative ion modes

	Toluene		Acetone	
Triterpenoid	(+) mode	(−) mode	(+) mode	(−) mode
Betulin	442 M^+•^	441 [M-H]^−^	425 [M + H-H_2_O]^+^	–
Lupeol	426 M^+•^	–	409 [M + H-H_2_O]^+^	–
Betulinic acid	456 M^+•^	455 [M-H]^−^	439 [M + H-H_2_O]^+^	455 [M-H]^−^
Betulonic acid^a^	454 M^+•^, 455 [M + H]^+^	453 [M-H]^−^	455 [M + H]^+^, 437 [M + H-H_2_O]^+^	453 [M-H]^−^
Allobetulin^a^	442 M^+•^, 443 [M + H]^+^	–	443 [M + H]^+^, 425 [M + H-H_2_O]^+^	–
Allobetulone	441 [M + H]^+^	–	441 [M + H]^+^	–

^a^When toluene was used as the dopant, the molecular ion (M^+.^) and protonated molecule ([M + H]^+^) were observed as main ions at intensity of approx. 1:1

In positive ion mode, all the studied triterpenoids were detected when toluene was used as the dopant. The most intense signals were observed for betulin, lupeol, and betulinic acid, which were detected as M^+•^ ions at *m*/*z* 442, 426, and 456, respectively. Betulonic acid showed M^+•^ at *m*/*z* 454 and [M + H]^+^ at *m*/*z* 455, and allobetulin was detected as M^+•^ at *m*/*z* 442 and as [M + H]^+^ at *m*/*z* 443. Allobetulone showed [M + H]^+^ at *m*/*z* 441. With acetone as the dopant, betulin, lupeol, and betulinic acid were detected as [M + H-H_2_O]^+^ fragment ions at *m*/*z* 425, 409, and 439, respectively [32, 33]. Betulonic acid, allobetulin, and allobetulone showed [M + H]^+^ at *m*/*z* 455, 443, and 441, respectively. Additionally, a minor fragment ion of [M + H-H_2_O]^+^ was observed with betulonic acid at *m*/*z* 437 [34]. A fragment ion of allobetulin was detected at *m*/*z* 425, which is probably due to the water loss as well. In an earlier study by Rhourri-Frih et al., betulin, lupeol, and betulinic acid were all detected as [M + H-H_2_O]^+^ in positive APPI-LC-MS with toluene as the dopant [33]. The reason for the different base peaks in ref. [33] and our DAPPI-MS measurements with toluene as the dopant is likely the LC solvent used on ref. [33], which takes part in the ionization process, and causes the protonation of the analytes [35].

In negative ion mode, abundant signals of [M-H]^−^ were detected with betulinic acid at *m*/*z* 455 [34] and with betulonic acid at *m*/*z* 453 [34] with both toluene and acetone. With toluene, betulin showed a minor [M-H]^−^ at *m*/*z* 441, but was not detected with acetone as the dopant. Lupeol, allobetulin, and allobetulone were not detected in negative ion mode.

Positive ion mode with toluene dopant was chosen for the DAPPI-MS experiments, because all the triterpenoids were detected in positive ion mode, and with toluene all the triterpenoids were detected mainly as molecular ions or as protonated molecules. Product ion spectra for the main triterpenoid ions were collected with positive ion μAPPI with toluene dopant, and the detected main ions are presented in the ESM in Table S1.

### Investigation of *B*. *pendula* bark fractions by DAPPI-MS

Four fractions of *B*. *pendula* bark samples from three different tree heights (mature stem (M), transition zone (T), and young apical stem (Y)) were analyzed with DAPPI-MS to distinguish the different chemical profiles of the bark fractions. Principal component analysis (PCA) was used in the data processing to get an overview of the differences between the fractions and the stem heights. The samples were named based on the tree height and the bark fraction. For example, MF1 corresponds to mature stem fraction 1. Three replicate DAPPI-MS measurements were averaged from each sample, and all the spectra were background subtracted. In the PCA calculations, the observations were the measured *m*/*z* values of the ions in the mass spectra, i.e., the detected ions. The ions that were included in the data processing were observed in all the three replicates, and they all had signal-to-noise ratios (S/N) > 3. The variables were the intensities of the corresponding ions.

**Fig. 4:**
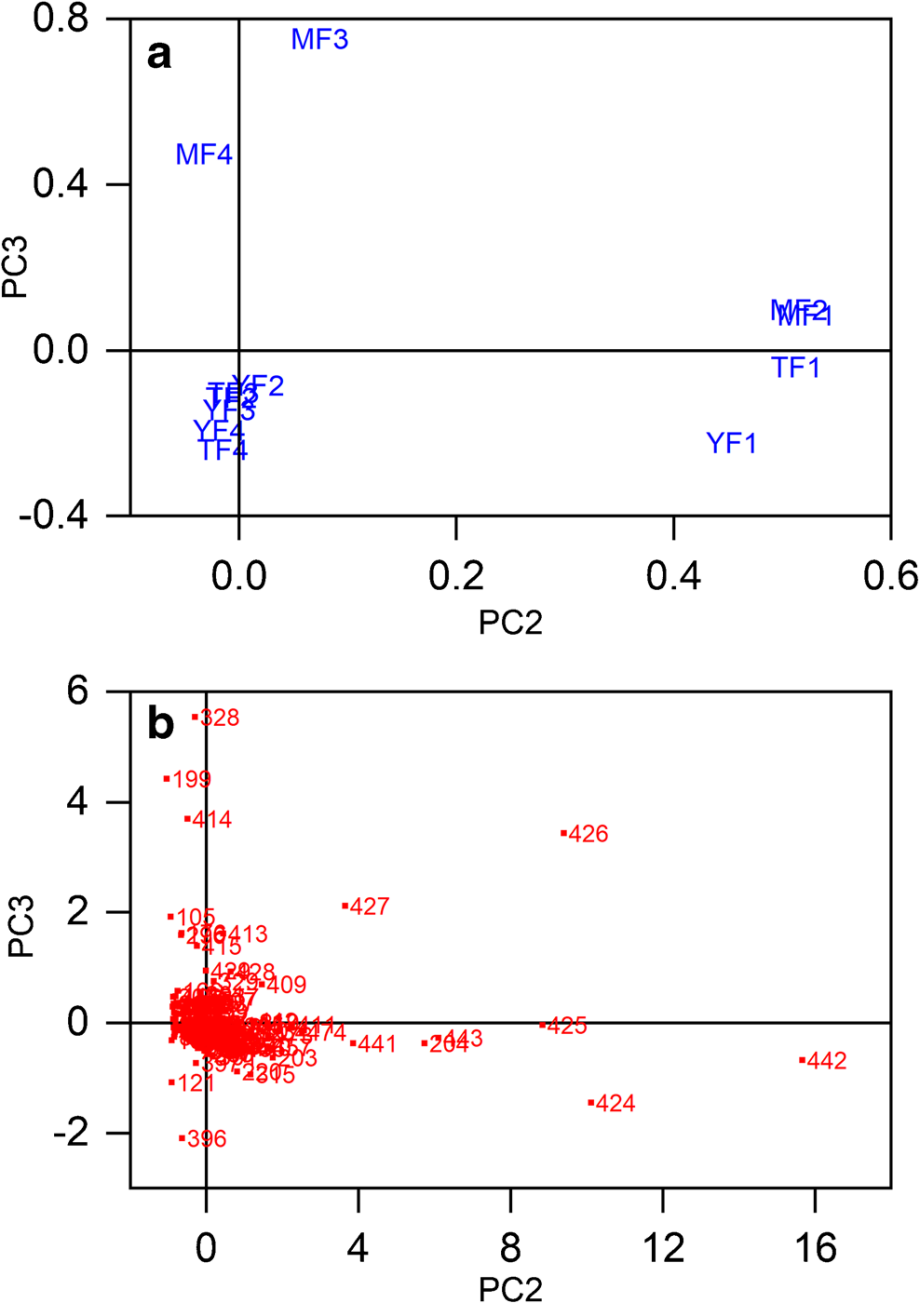
PCA calculation results of DAPPI-MS analysis of *B*. *pendula* bark fractions: **a** loadings plot showing the correlations between the chemical profiles of the samples from all the four fractions collected from three tree heights: mature stem (MF1–MF4), transition zone (TF1–TF4), and young apical stem (YF1–YF4), and **b** the score plot of the selected *m*/*z* peaks

Three principal components (PC) were calculated, and they covered approximately 88% of the variance in the data. Correlations of the chemical profiles of the fractions are visualized in the PCA plots in Fig. 4. The loadings plot (Fig. 4a) shows the correlation between the mass spectra of the samples YF1, TF1, MF1, and MF2 in the right-hand side of the figure. Additionally, MF3 and MF4 mass spectra correlate with each other, as do the spectra from samples TF2, TF3, TF4, YF2, YF3, and YF4 on the lower left-hand side of the loadings plot. The loadings plot shows that all the mature stem samples are separated to the positive side of the *y*-axis. However, the chemical profiles of MF1 and MF2 are different from MF3 and MF4, as they are aligned so far away from each other.

The score plot in Fig. 4b shows which ions contribute most in the separation of the chemical profiles of the samples. PC2 separates the ions at *m*/z 204, 424, 425, 426, 427, 441, 442, and 443 clearly to the positive side of the *x*-axis, and they have a great influence on the separation of the chemical profiles of the samples YF1, TF1, MF1, and MF2 from the rest of the samples. These ions are found in all F1 samples and the MF2 sample with high abundances, and they originate most likely from triterpenoids, as presented in Table 2. PC3 separates the ions at *m*/*z* 199, 328, and 414 to the upper left-hand side corner and the ions at *m*/*z* 105, 176, 296, 413, and 415 on the positive side of the *y*-axis, as shown in Fig. 4b. All these ions contribute to the alignment of samples MF3 and MF4 along the positive side of the *y*-axis in the loadings plot (Fig. 4a), and thus the separation of MF3 and MF4 samples from the rest of the samples. PC3 separates the ions at *m*/*z* 121 and 396 to the lower left-hand side of the score plot. These ions, especially the ion at *m*/*z* 396, are the main ions observed particularly in the mass spectra of samples F3 and F4, and they contribute to the separation of the young apical stem samples YF2, YF3, and YF4, and the transition zone samples TF2, TF3, and TF4 from the rest of the samples. The identities of the ions that affect the sample separations are discussed below.

**Table 2:** The triterpenoids observed in the DAPPI-MS studies of *B*. *pendula* bark fractions. The detected ion, main MS^2^ product ions, suggested ion identity, and level of identification are presented

Detected ion	DAPPI-MS^2^ main product ions	Tentative identification	Reference^a^	Level of identification^b^
442	424, 411, 427, 318, 189, 220, 203, 234	Betulin M^+•^	–	1
426	204, 411, 189, 218, 383, 408	Lupeol M^+•^	–	1
441	423, 411	Allobetulone [M + H]^+^	–	1
443	425	Allobetulin [M + H]^+^	–	1
424	409, 381, 189	[M-H_2_O]^+^ fragment of betulin M^+•^	–	2b
425	407, 217, 189, 191, 205, 203, 201, 177, 245	[M + H-H_2_O]^+^ fragment ion from betulin or allobetulin [M + H]^+^	[32–34] (for betulin)	3
454	190, 191, 439, 436, 410, 408, 248	Betulonic acid M^+•^	–	1
455	437, 409	Betulonic acid [M + H]^+^	[34]	1
456	438, 441, 248, 410, 234	Betulinic acid M^+•^	–	1
457	439, 411, 191	Betulinic acid [M + H]^+^	[32]	1

^a^Reference not found is marked with -

^b^The levels of identification as presented in ref. [36]. 1 = confirmed identification based on MS^2^ spectra and GC-MS measurements of the standard compounds and the samples; 2b = probable structure, identification based on MS^2^ spectra of the detected ions and standard compounds, ionization behavior of the compounds, and the experimental context; 3 = tentative structure, identification based on MS^2^ spectra of the detected ions and standard compounds, ionization behavior of the compounds, and the experimental context, but one exact structure is uncertain

**Fig. 5:**
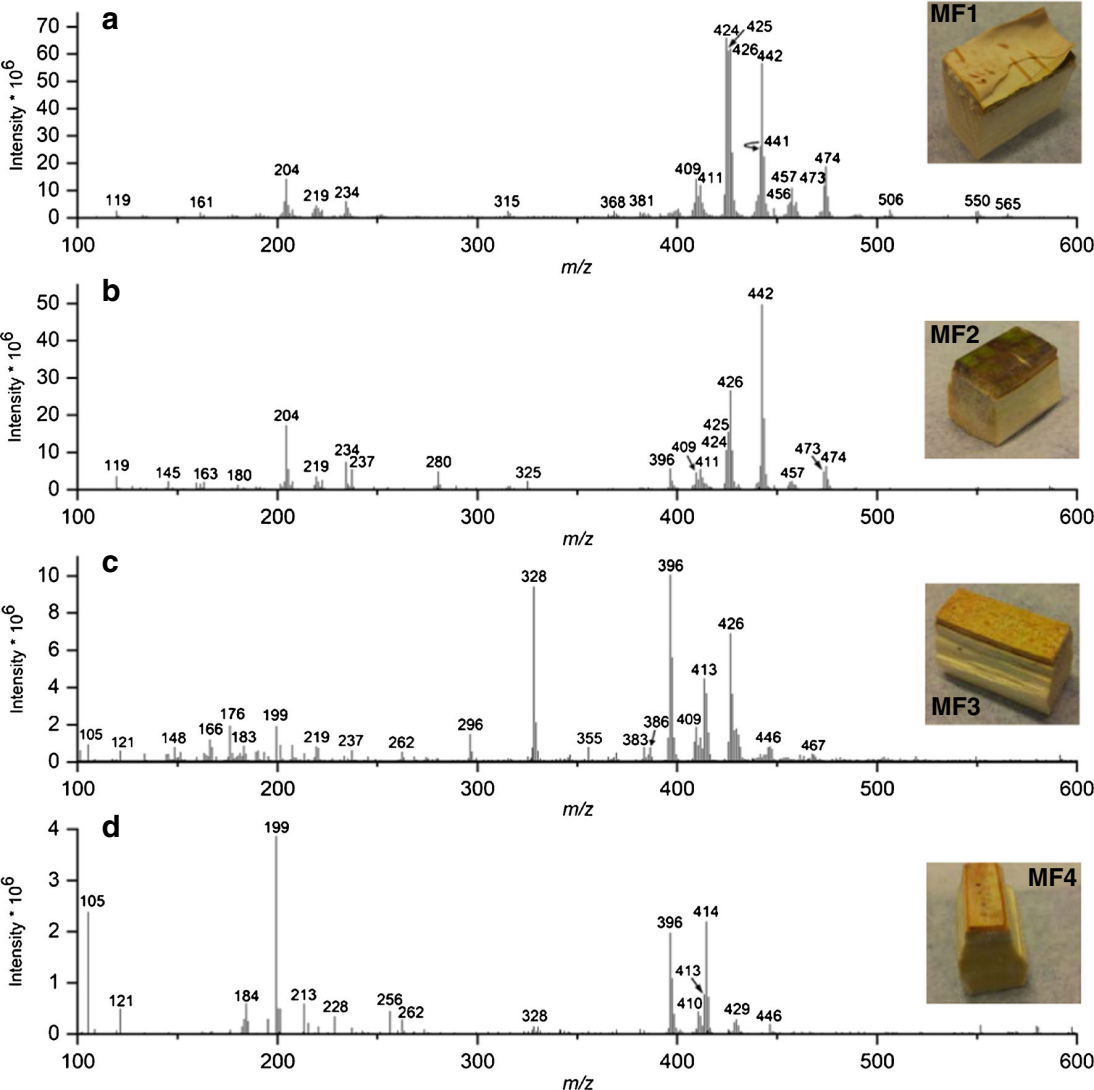
The DAPPI mass spectra of *B*. *pendula* MF1–MF4 in positive ion mode using toluene as the dopant: **a** MF1, **b** MF2, **c** MF3, and **d** MF4. Photos of the bark samples are also presented

As mentioned, the ions separated by PC2 originate mainly from triterpenoids that are abundant in the phellem of the bark in all the developmental stages of the stem. Figure 5 presents the DAPPI mass spectra and photos of the bark samples from the mature stem. The mass spectra show clearly that the chemical profiles of the fractions MF1–MF4 are different from each other. The triterpenoids are abundant in MF1 and MF2 in Fig. 5a, b, respectively, in the mass range of approximately 400–480 Da. The triterpenoids are less abundant in MF3, and completely absent in MF4. In the young apical stem and the transition zone, the triterpenoids were present in F1 with high abundances, but only residues were observed in F2, and nothing in fractions F3 and F4. This observation was also reported in an earlier report for betulin and lupeol [9].

The triterpenoids identified from the bark samples and the level of identification [36] are presented in Table 2. The identification was done by comparing the DAPPI-MS^2^ spectra of the bark samples to the direct infusion μAPPI-MS^2^ (Table S1 in ESM) measurements of the triterpenoid standards. However, the high triterpenoid concentrations in F1 and F2 samples may have led to alternative ionization products like fragments, which were not observed in the μAPPI experiments of the triterpenoid standards with the toluene dopant. The DAPPI identification was supported by the MS^2^ measurements and GC-MS measurements made from the bark samples for the identification of betulin, lupeol, betulinic acid, betulonic acid, allobetulin, and allobetulone ions. For the ions at *m*/z 424 and 425, the identification was supported by the MS and MS^2^ data of the samples and the standards (Table 2 and ESM Table S2). Tentative origins for some of the high-intensity ions detected from the samples are also suggested in the text as they are supported by literature and/or the experimental data.

The ions at *m*/*z* 442 and 426 were identified as M^+•^ of betulin and lupeol, respectively. The ion at *m*/*z* 443 was thought to be a combination of allobetulin [M + H]^+^ and betulin M^+•^ C^13^ isotopes, and the ion at *m*/*z* 427 was presumed to be mainly the lupeol C^13^ isotope, since lupeol did not protonate in direct infusion μAPPI studies of triterpenoids (Table 1). The ion at *m*/*z* 441 was identified as the [M + H]^+^ of allobetulone. Betulinic acid was detected as M^+•^ at *m*/z 456 and betulonic acid as both [M + H]^+^ and M^+•^ at *m*/*z* 455 and *m*/z 454, respectively. The ion at *m*/*z* 204 (Fig. 5a, b) could be a fragment of lupeol, since it was the main product ion of lupeol M^+•^ in μAPPI MS^2^ experiments (ESM Table S1). The ion at *m*/*z* 424 was assumed to be the [M-H_2_O]^+^ fragment of betulin (ESM Table S1). It was ruled out to originate from allobetulin, since the DAPPI-MS^2^ experiments of the *m*/z 442 ion from the birch bark did not show the allobetulin fragment ion at *m*/*z* 371 (ESM Table S1), and as a whole, the MS^2^ spectrum of the ion at *m*/z 442 corresponded to betulin M^+•^ MS^2^ spectrum. Therefore, it was concluded that the bark samples did not show M^+•^ of allobetulin. The ion at *m*/*z* 425 was assumed to be the [M + H-H_2_O]^+^ of betulin [32–34] or allobetulin, as it was observed in the μAPPI experiments with acetone dopant for both compounds (Table 1 and ESM Table S2). The ions at *m*/z 409 and 411 were typical product ions for many of the triterpenoids (ESM Table S1). An intense group of ions related to the triterpenoids can be observed in Fig. 5a, b in the mass range of approx. 470–475. These ions are suggested to be the oxidation products of the triterpenoid acids.

The ions at *m*/z 105, 121, 176, 199, 296, 328, 396, 413, 414, and 415, presented in Fig. 5, which contribute to the separation of fractions F2–F4 of transition zone and young apical stem, and the samples MF3 and MF4 from all the F1 samples in the PCA (Fig. 4), were not identified, but they are suggested to be other plant metabolites present in the bark. For example, according to literature, the phytosterol β-sitosterol (molecular weight 414 Da) is present in the birch bark [37, 38], and was detected in GC-MS experiments conducted from samples from the same trees as in this study (unpublished results). β-Sitosterol shows M^+•^ at *m*/*z* 414, and a [M-H_2_O]^+^ fragment at *m*/z 396 in electron ionization-mass spectrum [39]. Therefore, the ions at *m*/*z* 396 and 414 (Fig. 5d) are suggested to originate from β-sitosterol. Additionally, the natural arylbutanoid glycoside rhododendrin with molecular weight of 328 is present in the inner bark of *B*. *pendula* [40, 41], and therefore the ion at *m*/*z* 328 in Fig. 5c, d could be due to the M^+•^ of rhododendrin. In negative ion electrospray ionization (ESI), rhododendrin has been detected as [M-H]^−^ at *m*/*z* 327 [41], and in positive ion ESI as [M + 23]^+^ at *m*/*z* 351 [40].

It can be concluded that the triterpenoids dominate the chemical profile of the *B*. *pendula* bark fractions F1 and MF2, which contain a high amount of triterpenoids, including, e.g., betulin, lupeol, betulinic acid, and betulonic acid, even though the concentrations of the triterpenoids decrease in the order of mature stem > transition zone > young apical stem [9]. Compared to F1, the other fractions in all the developmental stages lack triterpenoids. Instead, they are rich with other plant metabolites, such as β-sitosterol and rhododendrin.

### Analysis of lenticels of *B*. *pendula*, *A*. *glutinosa*, and *A*. *incana* by DAPPI-MS

**Fig. 6:**
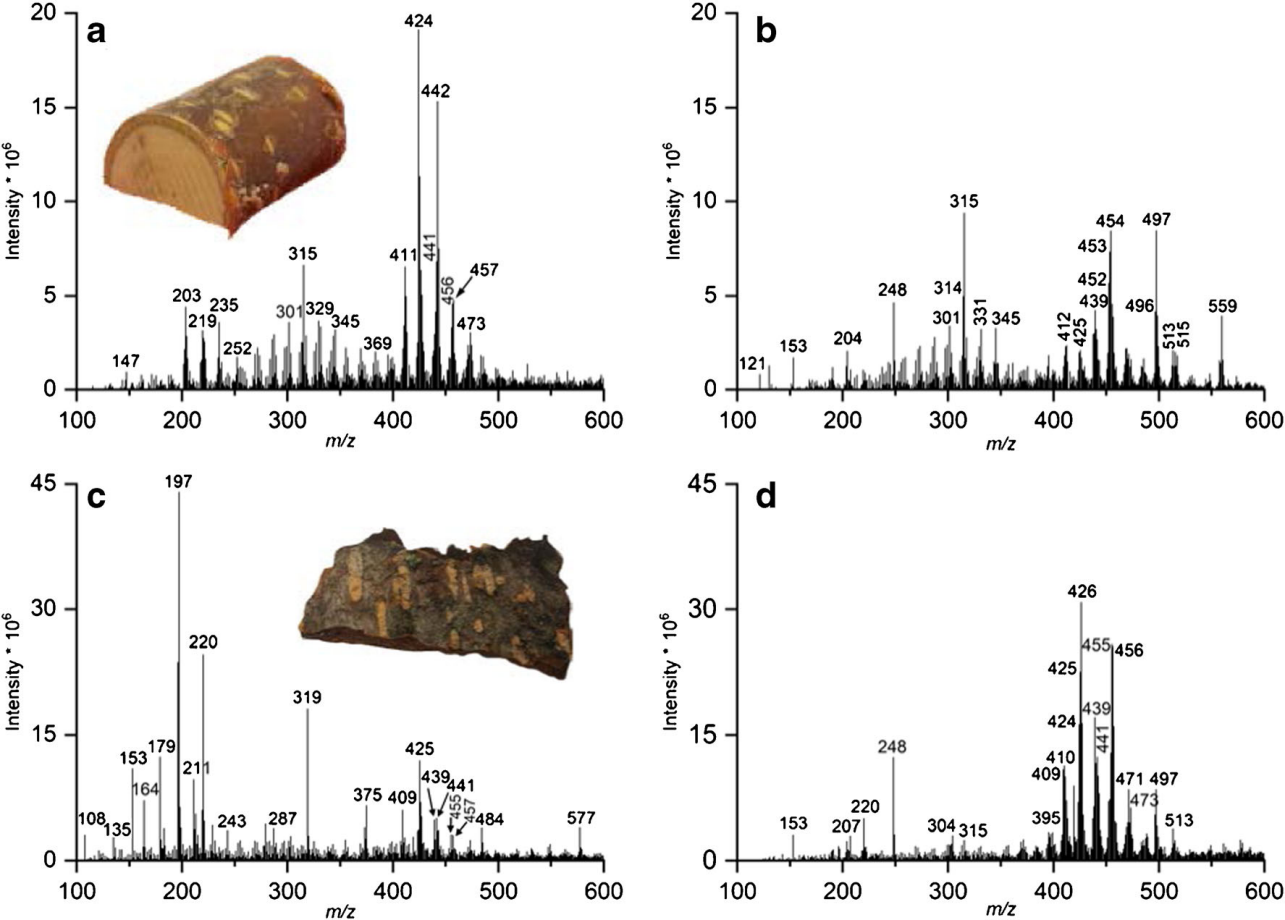
DAPPI mass spectra of *B*. *pendula* YF1 and *A*. *glutinosa* MF1 analysis of lenticels and the surrounding surface and example photographs of the samples. The measurements were done in positive ion mode using toluene as the dopant. **a***B*. *pendula* surrounding surface analysis, **b***B*. *pendula* lenticel analysis, **c***A*. *glutinosa* surrounding surface analysis, and **d***A*. *glutinosa* lenticel analysis

DAPPI-MS was used to study the lenticels and the surrounding surface from the F1 of the bark of *B*. *pendula*, *A*. *glutinosa*, and *A*. *incana*. Figure 6 presents the mass spectra from YF1 of *B*. *pendula* (Fig. 6a, b) and MF1 of *A*. *glutinosa* (Fig. 6c, d) samples, and shows photographs of the samples. Both bark samples contained clear light-colored lenticels surrounded by darker surface, which in the young apical stem surface of *B*. *pendula* is brown and in *A*. *glutinosa* dark gray. For both tree species, it was found that the lenticels and the surrounding tissue contain distinct chemical compositions (Fig. 6).

For *B*. *pendula*, the F1 surface showed a high amount of triterpenoids (Fig. 6a), as discussed above. The betulin M^+•^, its propable fragment [M-H_2_O]^+^, and allobetulone [M + H]^+^ at *m*/*z* 442, 424, and 441, respectively, were observed with high abundances in the tissue surrounding the lenticels. In the lenticels, betulinic and betulonic acids, detected as [M + H]^+^ at *m*/*z* 456 and 455, respectively, were the main triterpenoids (Fig. 6b) at higher abundance than in the surrounding tissue (Fig. 6a). The ion at *m*/z 439 was assumed to be a combination of fragments of betulonic and betulinic acids, respectively (ESM Table S1). Instead, the intensities of betulin and lupeol M^+•^ ions at *m*/*z* 442 and 426, respectively, in the lenticels were negligible compared to the surrounding surface.

For *A*. *glutinosa*, the surface of the phellem (excluding the lenticels) contained only a small amount of triterpenoids (Fig. 6c) and for example the betulin M^+•^ abundance was approx. 5% of the intensity of what was measured from the YF1 samples of *B*. *pendula*. Instead, the lenticels of *A*. *glutinosa* showed high abundancies for triterpenoids in the *m*/z range of approx. 400–480 in Fig. 6d. The highest triterpenoid intensity was measured for lupeol M^+•^ at *m*/*z* 426. The intensities of betulonic acid [M + H]^+^ and betulinic acid M^+•^, at *m*/*z* 455 and 456, respectively, were approximately five times higher in the lenticels than in the surface surrounding the lenticels. The ions at *m*/*z* 409 and 410 are possibly the fragments of betulonic acid [M + H]^+^ and M^+•^ ions (ESM Table S1), and the ion at *m*/*z* 439 the fragment of betulonic and betulinic acids, as described previously. The intense ion at *m*/*z* 248 may be a fragment of both betulonic and betulinic acid M^+•^ (ESM Table S1), and it is observed in the mass spectrum of the lenticel measurements from both *B*. *pendula* and *A*. *glutinosa* (Fig. 6b, d). Again, the [M + H-H_2_O]^+^ ion at *m*/*z* 425 is most likely to originate from betulin [34] or allobetulin [M + H]^+^ (Table 1 and ESM Table S2). The allobetulone [M + H]^+^ ion at *m*/*z* 441 was also detected. The higher abundancies of betulinic and betulonic acids in the lenticels compared to the surrounding bark surface may be due to the efficient oxidizing reaction of the betulin taken place at the lenticels, which act as gas exchange channels between the atmosphere and the inner parts of the tree.

The color of the lenticels in the *A*. *incana* F1 surface was much lighter than in the lenticels on *A*. *glutinosa*, almost indistinguishable from the surrounding bark area. The surrounding surface contained clear light gray and dark gray areas (see ESM, Fig. S2). The light and dark areas, as well as the lenticels, were analyzed separately by DAPPI-MS, and the mass spectra are presented in the ESM in Fig. S3. Triterpenoids were detected from the lenticels (ESM Fig. S3a), but the intensities were much lower than in the *A*. *glutinosa* F1 samples. The main triterpenoid detected was the lupeol M^+•^ at *m*/*z* 426. Betulonic acid [M + H]^+^ and betulinic acid M^+•^ were detected at *m*/*z* 455 and 456, respectively, as well as the ion at *m*/*z* 439, most likely originating from both betulonic and betulinic acids. None of the triterpenoids was detected from the surrounding tissue. However, the mass spectra of the light (ESM Fig. S3b) and dark gray (ESM Fig. S3c) areas were very distinct from each other. In the light area, the two most abundant ions were observed at *m*/*z* 196 and 220, but in the dark gray area two intense ions appeared at *m*/*z* 345 and 467. However, these ions remain unidentified.

### LAAPPI-MSI of *B*. *pendula* lenticels

DAPPI utilizes a wide and continuous solvent plume for sampling, and thus the technique’s ability to locate compounds could be questioned. LAAPPI, instead, enabled spatially precise sampling of the F1 surface. Here, LAAPPI-MSI was used to study the distributions of all the previously detected triterpenoids at *B*. *pendula* F1 lenticel regions. The distribution of betulinic acid was of particular interest, as DAPPI-MS showed betulinic acid to be more abundant at the F1 lenticel regions than the other areas on the F1 surface.

Acetone was chosen as the dopant for the LAAPPI-MSI measurements, because it drives the ionization process toward formation of protonated molecules, and this simplifies the surface screening analysis. Moreover, DAPPI-MS and μAPPI-MS experiments with acetone showed that most triterpenoids can be detected with at least one characteristic mass peak.

**Fig. 7:**
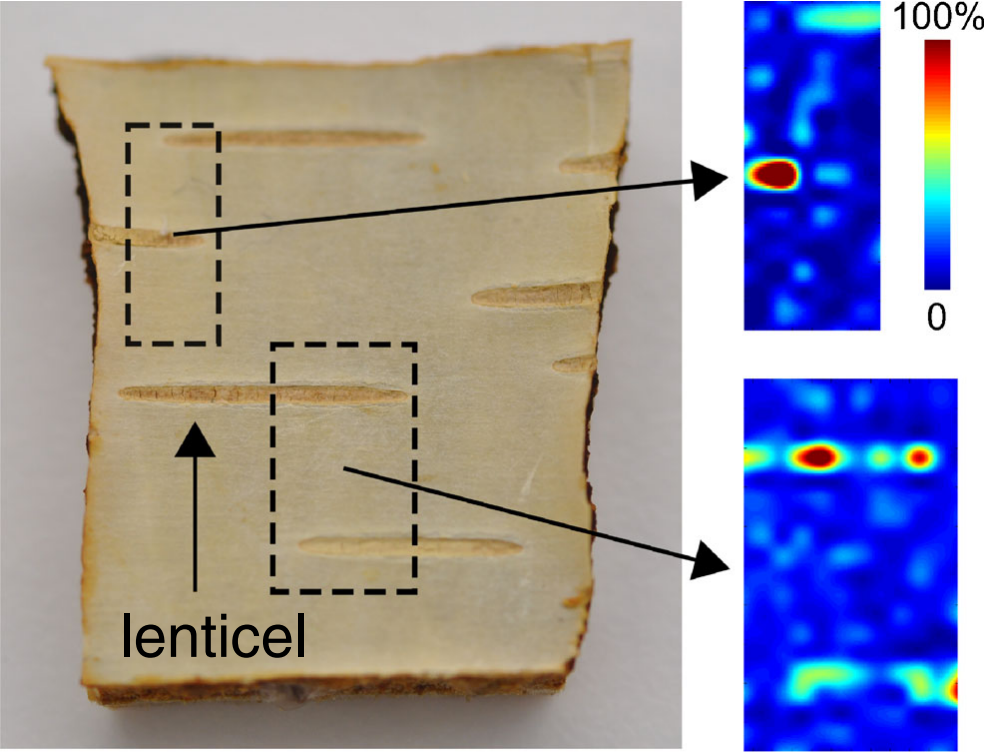
The distribution of betulinic acid in MF1 fraction of *B*. *pendula* measured by LAAPPI-MSI. Red and blue colors indicate high and low abundancies, respectively. The heat map images show that betulinic acid is clearly more abundant in the lenticel regions of the analyzed sample areas (dashed boxes)

LAAPPI-MS detected all the same triterpenoids as DAPPI-MS (Table 1). Furthermore, the created heat map images show clear betulinic acid distribution patterns around the F1 lenticel regions as presented in Fig. 7. Betulinic acid was found to be much more abundant at the lenticel regions of F1 (deep red color) compared to the surrounding tissue, as was observed with DAPPI-MS as well. All other triterpenoids were more uniformly distributed in F1, and no clear distribution patterns were detected in addition to the compounds’ lower abundances at the lenticel regions.

## Conclusions

In this work, we applied ambient MS for the first time to the rapid analysis of plant metabolites directly from tree surface. DAPPI proved to be an efficient method for ionizing triterpenoids. Triterpenoids and other plant metabolites were detected from *B*. *pendula* bark fractions collected from three different developmental stages of the tree. The DAPPI-MS analysis showed unique chemical patterns for all the four studied bark fractions. The first two fractions of the mature stem (phellem and phelloderm tissues) were found to be rich with triterpenoids, but from the transition zone and the young apical stem, only the first fraction (phellem) of the bark was abundant with triterpenoids. The triterpenoids with the highest abundancies were betulin and lupeol. DAPPI-MS was also feasible for the screening of lenticels and the surrounding tissue from *B*. *pendula* and *A*. *glutinosa* F1 samples. The lenticels were shown to contain higher amounts of betulonic and betulinic acids than the surrounding tissue. The mature stem F1 of birch was also analyzed with LAAPPI-MSI, and the betulinic acid was detected with higher abundancy from the lenticels than from to the surrounding tissue. Thus it can be concluded that the LAAPPI-MSI measurements verified the DAPPI-MS results, and the sampling accuracy of DAPPI-MS was suitable for a proper analysis of lenticel-sized regions and the technique could be beneficial in rapid screening analysis of tree samples.

## Electronic supplementary material

ESM 1 (PDF 314 kb)
