# Supplementary material for: Chemical profiles of birch and alder bark by ambient mass spectrometry
Source: Anal Bioanal Chem. 2019 Oct 23;411(28):7573–83. doi: 10.1007/s00216-019-02171-9 (PMC6875546; doi:10.1007/s00216-019-02171-9)
Supplement: Supplementary file 1 — (PDF 314 kb) [file 216_2019_2171_MOESM1_ESM.pdf]

Analytical and Bioanalytical Chemistry

Electronic Supplementary Material

Chemical profiles of birch and alder bark by ambient mass spectrometry

Riikka-Marjaana Räsänen, Juha-Pekka Hieta, Juha Immanen, Kaisa Nieminen, Raisa Haavikko, Jari Yli-Kauhaluoma, Tiina J. Kauppila

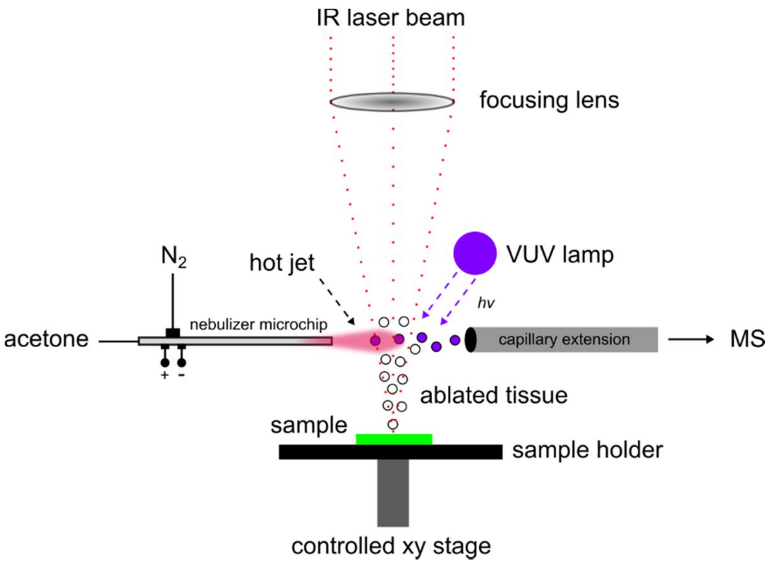

Fig. S1 A schematic picture of the LAAPPI-MSI setup

Table S1 Observed triterpenoid precursor ions and their MS^2 and MS^3 (marked with *) fragments in direct infusion µAPPI experiments, with toluene as the dopant. The main peak of the compound was chosen as the precursor ion. Nebulizer gas (N_2) flow was 180 mL / min, microchip heated nebulizer heating power was 4.5 W, triterpenoid standard concentrations were 10 µM in toluene infused with 5 µL/min flow rate. Experiments were performed in positive ion mode. Only product ions with ≥ 10% intensities compared to the base peak intensity in the MS^2 spectrum, and ≥ 20% in the MS^3 spectrum are presented

Triterpenoid	Precursor ion and relative intensity (%) from the base peak	Observed MS^2 fragments and relative intensities (%) from the base peak
Betulin	442 $M^{+\bullet}$ (7)	424 (100), 411 (93), 427 (68), 318 (41), 203 (29) 189 (29), 235 (23), 234 (20), 220 (20), 207 (19), 381 (17), 399 (13), 206 (13), 190 (11), 409 (11), 413 (11), 205 (10)
	424* (13)	189 (100), 381 (90), 409 (82), 204 (38), 202 (31), 136 (30), 190 (29), 406 (27), 393 (23), 187 (22), 216 (21)
Lupeol	426 $M^{+\bullet}$ (5)	204 (100), 411 (98), 218 (89), 189 (66), 313 (36), 203 (35), 383 (34), 300 (34), 207 (22), 234 (21), 408 (20), 161 (16), 175 (16), 220 (15), 257 (14), 272 (14), 315 (13), 316 (13), 205 (13), 286 (12), 162 (12), 147 (12), 187 (11), 206 (11), 191 (11), 190 (10), 148 (10)
Betulinic acid	456 $M^{+\bullet}$ (7)	438 (100), 234 (50), 248 (47), 189 (42), 316 (32), 220 (31), 410 (27), 207 (24), 395 (22), 302 (22), 441 (20), 202 (19), 187 (18), 203 (16), 191 (13), 219 (12), 423 (12), 190 (11)
Betulonic acid	454 $M^{+\bullet}$ (4)	410 (100), 408 (41), 248 (34), 190 (22), 439 (17), 205 (15), 436 (14), 235 (11)
	455 $[M+H]^+$ (5)	437 (100), 409 (42), 177 (23), 411 (18), 343 (13), 391 (10), 259 (10)
Allobetulin	442 $M^{+\bullet}$ (4)	424 (100), 411 (27), 220 (24), 371 (23), 149 (18), 399 (14), 205 (14), 189 (11)
	443 $[M+H]^+$ (5)	425 (100), 413 (31), 191 (30)
Allobetulone	441 $[M+H]^+$ (6)	423 (100), 411 (65), 329 (13), 177 (12), 245 (11)
	440 $M^{+\bullet}$ (8)	422 (100), 410 (93), 369 (47), 220 (41), 204 (33), 148 (17), 397 (16), 199 (15), 425 (13), 205 (12), 202 (10), 391 (10), 217 (10)

Table S2 Observed betulin and allobetulin precursor ions and their MS^2 fragments in direct infusion µAPPI experiments, with acetone as the dopant. Nebulizer gas (N_2) flow was 180 mL / min, microchip heated nebulizer heating power was 4.5 W, triterpenoid standard concentrations were 10 µM in acetone, infused with 5 µL/min flow rate. Experiments were performed in positive ion mode. Only product ions with ≥ 10% intensities compared to the base peak intensity in the MS^2 spectrum are presented

Triterpenoid	Precursor ion and relative intensity (%) from the base peak	Observed MS^2 fragments and relative intensities (%) from the base peak
Betulin	425 (4)	407 (100), 219 (20), 283 (18), 217 (15), 245 (15), 201 (15) 255 (14), 269 (14), 191 (14), 257 (14), 273 (12), 227 (11), 397 (11), 215 (10)
Allobetulin	425 (6)	407 (100), 247 (63), 245 (60), 191 (50), 397 (29), 229 (26), 217 (24), 301 (21), 283 (18), 203 (17), 177 (14), 227 (14), 273 (12), 257 (12), 179 (11), 275 (11), 173 (11), 395 (10)

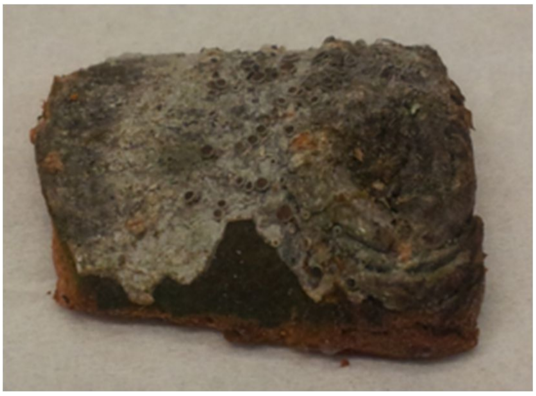

Fig. S2 *A. incana* F1 sample (mature stem)

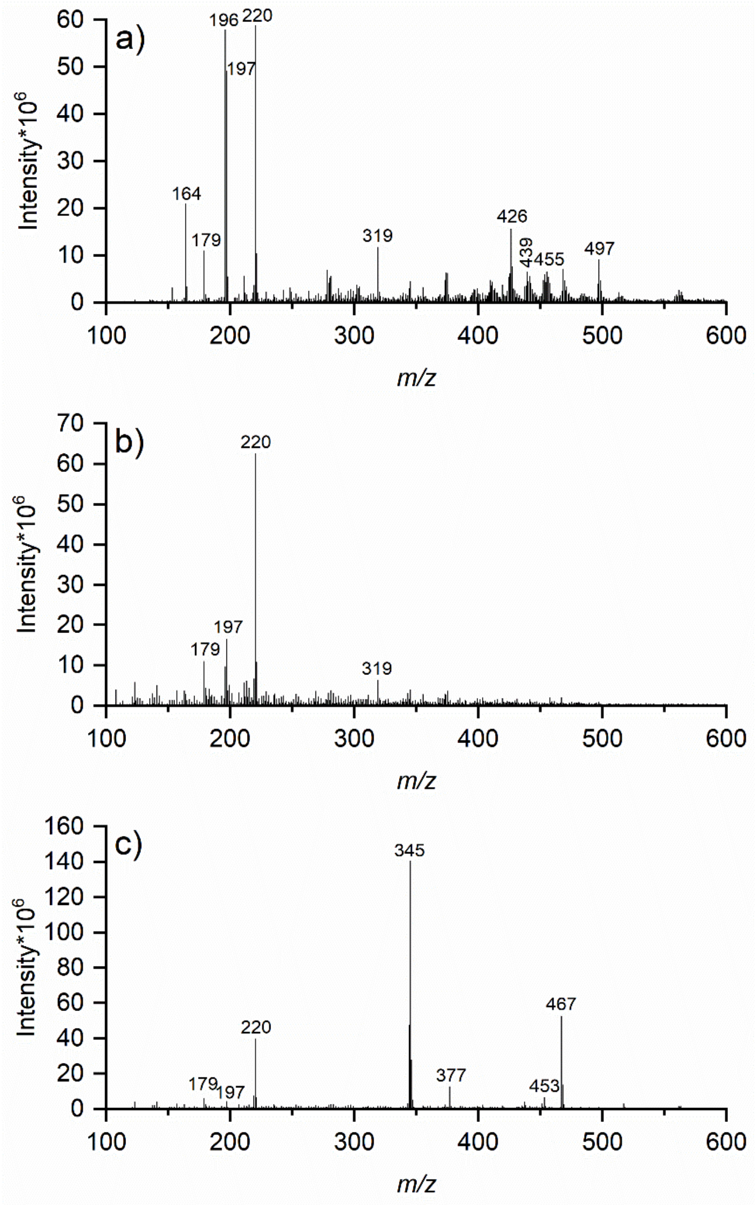

Fig. S3 *A. incana* DAPPI mass spectra from a) lenticel, b) light grey surface, and c) dark grey surface
